# Oligoribonucleotide interference-PCR-based methods for the sensitive and accurate detection of *KRAS* mutations

**DOI:** 10.1093/biomethods/bpae071

**Published:** 2024-10-01

**Authors:** Hiroaki Fujita, Toshitsugu Fujita, Keinosuke Ishido, Kenichi Hakamada, Hodaka Fujii

**Affiliations:** Department of Gastroenterological Surgery, Hirosaki University Graduate School of Medicine, 5 Zaifu-cho, Hirosaki, 036-8562, Aomori, Japan; Department of Biochemistry and Genome Biology, Hirosaki University Graduate School of Medicine, 5 Zaifu-cho, Hirosaki, 036-8562, Aomori, Japan; Department of Gastroenterological Surgery, Hirosaki University Graduate School of Medicine, 5 Zaifu-cho, Hirosaki, 036-8562, Aomori, Japan; Department of Gastroenterological Surgery, Hirosaki University Graduate School of Medicine, 5 Zaifu-cho, Hirosaki, 036-8562, Aomori, Japan; Department of Biochemistry and Genome Biology, Hirosaki University Graduate School of Medicine, 5 Zaifu-cho, Hirosaki, 036-8562, Aomori, Japan

**Keywords:** ORNi-PCR, ddPCR, real-time PCR, gene mutation, KRAS

## Abstract

Pancreatic cancer is an aggressive malignancy with a poor prognosis. Single-nucleotide mutations in the *KRAS* gene are detected in the majority of patients with pancreatic ductal adenocarcinoma (PDAC), the most common type of pancreatic cancer. Identifying *KRAS* mutations by liquid biopsy could be effective for detecting *de novo* and recurrent PDAC; however, sensitive and accurate detection remains challenging. We examined the utility of oligoribonucleotide interference-PCR (ORNi-PCR) followed by real-time PCR or droplet digital PCR (ddPCR) for detecting *KRAS* single-nucleotide mutations by liquid biopsy. A model of cell-free DNA was used to demonstrate that the ORNi-PCR-based methods are more sensitive and accurate for detecting *KRAS* mutant DNA than conventional real-time PCR or ddPCR. ORNi-PCR-based methods could be useful for early detection of *de novo* and recurrent PDAC by liquid biopsy for cancer diagnosis.

## Introduction

Pancreatic cancer is an aggressive malignancy with a poor prognosis. Early detection of pancreatic cancer is important for the clinical management of this malignancy. Imaging methods and biomarker testing using blood specimens are widely used for the diagnosis of pancreatic cancer; however, these methods show limited sensitivity [1]. Furthermore, pancreatic cancer often remains undetected until the advanced stages because of the absence of specific clinical symptoms, underscoring the need to develop more sensitive methods for the early detection of this malignancy.

Pancreatic ductal adenocarcinoma (PDAC), a highly aggressive lethal malignancy, accounts for >90% of pancreatic cancers [2, 3]. Several gene mutations are found in PDAC, among which *KRAS* mutations are present in most PDAC cases [2, 4]. *KRAS* mutations occur predominantly at Glycine 12 (G12) and Glycine 13 (G13) (e.g., G12D/V/R, G13D) positions, accounting for >98% of all mutations [4]. Thus, detection of these *KRAS* mutations could be of value for the early detection of *de novo* and recurrent PDAC. Liquid biopsy using blood specimens is an optimal method because of its low invasiveness, and it could be applied for the development of sensitive and accurate methods for detecting *KRAS* mutations, which are urgently needed.

Real-time PCR using dual-labeled probes is used to detect target gene mutations [5]. Although real-time PCR is sensitive, the presence of large amounts of wild-type (WT) sequences in the sample DNA pool impairs amplification (detection) of a target nucleotide mutation. Real-time PCR can detect a target gene mutation by liquid biopsy if it occurs at a frequency of >1% [1% variant allele frequency (VAF)] [6]. Droplet digital PCR (ddPCR) is a sensitive method that can detect >0.01–0.001% VAF of a target gene mutation [6, 7]. However, detecting gene mutations with high accuracy while avoiding false positives remains challenging.

**Figure 1.**
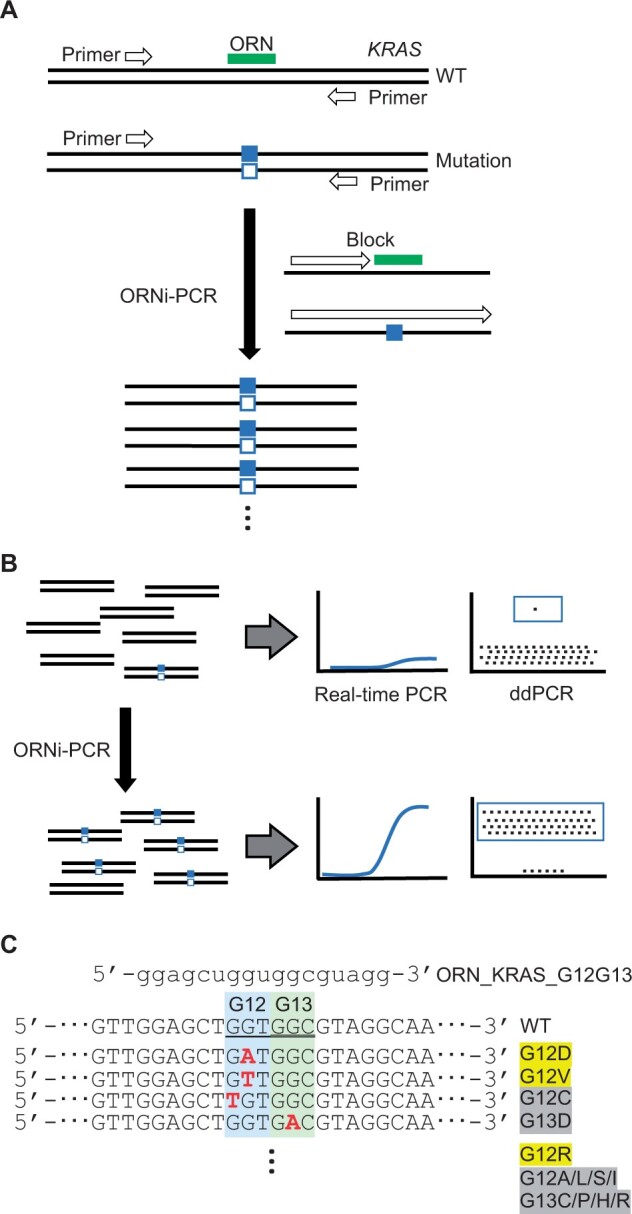
Schematic diagram of ORNi-PCR. (**A**) Schematic diagram of ORNi-PCR. ORNi-PCR can specifically amplify a target mutation sequence while suppressing amplification of wild-type (WT) sequences. Squares indicate nucleotide mutations. (**B**) Schematic diagram of ORNi-PCR followed by real-time PCR or droplet digital PCR (ddPCR). (**C**) The nucleotide sequence of an ORN targeting the human *KRAS* sequence. The forward *KRAS* sequences assessed in this study are shown. Major and minor mutations in PDAC are highlighted in yellow and gray, respectively.

PCR blockers can block amplification of WT sequences to specifically amplify a target gene mutation [8]. We previously identified an oligoribonucleotide (ORN) that can be used as a sequence-specific nucleotide blocker and developed a system called “ORN interference-PCR (ORNi-PCR)” (Fig. 1A) [9, 10]. Because of the specific amplification of a target gene mutation, ORNi-PCR followed by real-time PCR/ddPCR can detect a target gene mutation with high sensitivity and accuracy compared with conventional real-time PCR/ddPCR (Fig. 1B).

In this study, we examined the utility of ORNi-PCR followed by real-time PCR/ddPCR for detecting *KRAS* single-nucleotide mutations at the G12/G13 positions (hereafter referred to as “G12X DNA” or “G13X DNA”) using a liquid biopsy model. The results suggest that the ORNi-PCR-based methods are useful for early detection of PDAC by liquid biopsy.

## Materials and methods

### Oligonucleotides

An ORN was chemically synthesized by FASMAC (Tokyo, Japan). Primers and dual-labeled fluorescent probes were chemically synthesized by Eurofin Genomics (Tokyo, Japan). The ORN, primers, and probes are listed in Supplementary Table S1.

### Template DNA

Genomic DNA (gDNA) was extracted from a 15-μm section of each formalin-fixed paraffin-embedded (FFPE) cell sample [KRAS G12D Reference Standard, 5% (HD119, Horizon Discovery, Cambridge, UK) and EGFR Gene-Specific Multiplex Reference Standard (FFPE) 5% AF (HD300, Horizon Discovery)] using the Quick-DNA FFPE Kit (Zymo Research, Irvine, CA, USA). HD119, but not HD300, includes cells with the *KRAS* G12D mutation (5% of the total cells). The human *KRAS* WT sequence was amplified using hKRAS-F and hKRAS-R primers with the KRAS G12D Reference Standard, 50% (HD272, Horizon Discovery). The PCR amplicon was cloned using the Zero Blunt TOPO PCR Cloning Kit for Sequencing (450159, Thermo Fisher Scientific, Waltham, MA, USA) to construct the plasmid pCR4_hKRAS_WT (Supplementary Fig. S1). The nucleotide mutations corresponding to G12D, G12C, and G12V were introduced into pCR4_hKRAS_WT using the QuikChange Site-Directed Mutagenesis Kit (200519, Agilent, Santa Clara, CA, USA) to generate pCR4_hKRAS_G12D, pCR4-hKRAS_G12C, and pCR4-hKRAS_G12V, respectively. The human *KRAS* G13D sequence was amplified using hKRAS-F and hKRAS-R primers with gDNA extracted from a human colorectal carcinoma cell line, HCT116. The amplicon was cloned using the Zero Blunt TOPO PCR Cloning Kit for Sequencing to construct pCR4_hKRAS_G13D. Cell-free DNA (cfDNA) was extracted from 4 mL of the plasma fraction of a healthy donor (12271420, Cosmo Bio, Tokyo, Japan) using the QIAamp Circulating Nucleic Acid Kit (55114, QIAGEN, Hilden, Germany) with a modification (doubling the wash step with Buffer ACW2). NanoDrop One (ND-ONE-W, Thermo Fisher Scientific) was used to measure DNA concentration. The copy number of a target DNA can be calculated from the length and concentration of DNA.

### ORNi-PCR followed by real-time PCR or ddPCR

The reaction mixtures for ORNi-PCR, real-time PCR, and ddPCR with hKRAS-G12G13-F and hKRAS-G12G13-R primers are described in Supplementary Tables S2–S4. Thermal cycling was performed with the Mastercycler nexus gradient (6331000033, Eppendorf, Hamburg, Germany) or the CFX Connect Real-Time PCR Detection System (1855201J1, Bio-Rad, Hercules, CA, USA). Thermal cycling conditions are described in each figure. After ORNi-PCR, 1 μL of 100-fold-diluted ORNi-PCR sample was subjected to real-time PCR or ddPCR. For ddPCR, the fluorescence was detected using the QX200 Droplet Reader (1864003JA, Bio-Rad). QuantaSoft Version 1.7 (1864011JA, Bio-Rad) was used for data analysis.

### DNA sequencing analysis

ORNi-PCR products were subjected to DNA sequencing analysis (Eurofin Genomics). SnapGene Viewer (https://www.snapgene.com/snapgene-viewer) was used for data analysis.

## Results and discussion

### Specific amplification of *KRAS* single-nucleotide mutations by ORNi-PCR

**Figure 2.**
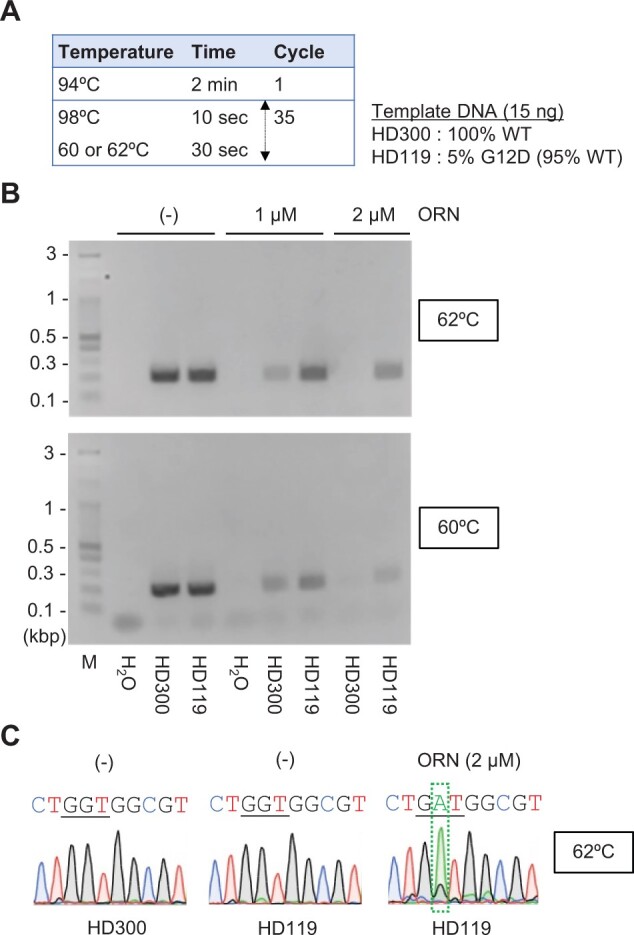
Detection of *KRAS* G12D DNA by ORNi-PCR. (**A** and **B**) Experimental conditions (**A**) and results of ORNi-PCR (**B**). M: molecular weight marker. DNA extracted from HD119, but not HD300, includes the *KRAS* G12D mutation (5% of VAF). (**C**) DNA sequencing analysis of ORNi-PCR amplicons shown in (**B**).

First, we designed an ORN for ORNi-PCR. As shown in Fig. 1C, an ORN complementary to the *KRAS* WT sequence around the G12/G13 positions can be used to enrich *KRAS* single-nucleotide mutations corresponding to the G12/G13 positions. Next, the optimal experimental conditions for ORNi-PCR were established using the designed ORN to specifically amplify *KRAS* mutant DNA. We first focused on G12D DNA (Fig. 1C) because it is the most frequent *KRAS* mutation in PDAC [4]. Following the optimization protocol for ORNi-PCR [10], the experimental conditions were optimized to specifically amplify G12D DNA while suppressing WT DNA (Fig. 2A). Because the reaction temperature (annealing plus elongation) and ORN concentration are critical for the optimization [10], we tested different temperatures and concentrations and found that 2 µM of the ORN and a temperature of 60–62°C for the annealing plus elongation step were optimal to amplify G12D DNA in the model gDNA (5% VAF of G12D) (Fig. 2B and C).

**Figure 3.**
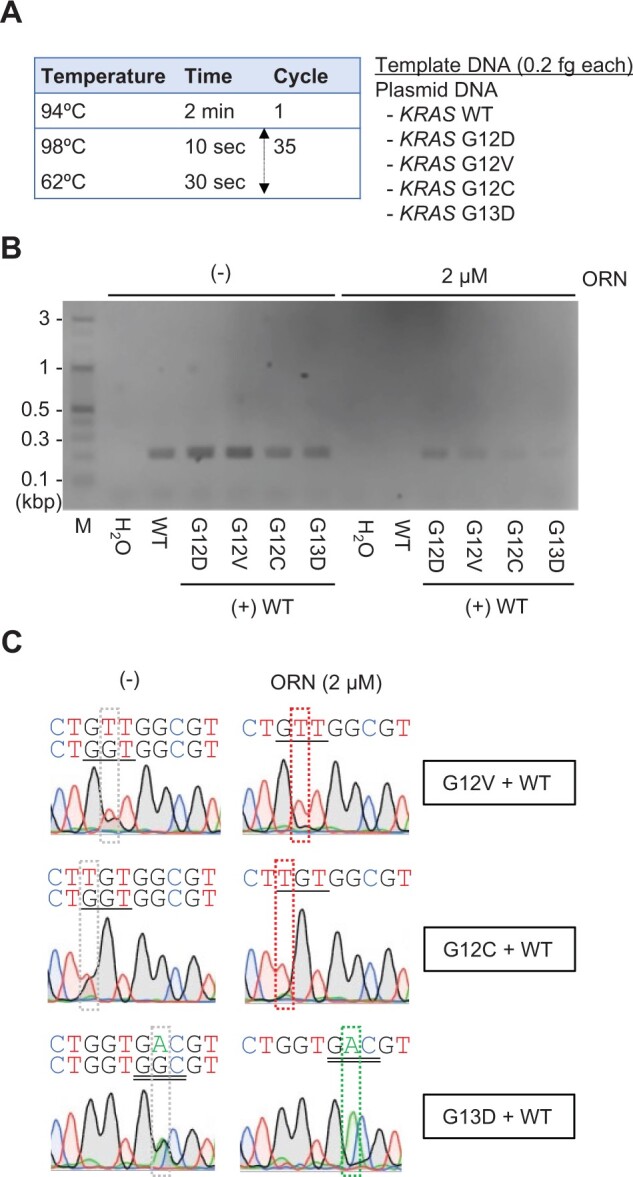
Detection of *KRAS* G12V/C and G13D DNA by ORNi-PCR. (**A** and **B**) Experimental conditions (**A**) and results of ORNi-PCR (**B**). M: molecular weight marker. (**C**) DNA sequencing analysis of ORNi-PCR amplicons shown in (**B**).

We next focused on another major mutation (G12V) and two minor mutations (G12C and G13D) found in PDAC (Fig. 1C). A mixture of plasmid DNA was used as the model (Fig. 3A and B) to confirm the optimal experimental condition (2 µM of the ORN and a temperature of 62°C for the annealing plus elongation step) for the specific amplification of *KRAS* mutant DNA and suppression of WT DNA. Because of the specific amplification of *KRAS* mutant DNA (Fig. 3B and C), this ORNi-PCR protocol was selected for specifically amplifying *KRAS* G12X and G13X mutations in a sample DNA pool.

### Detection of *KRAS* G12V DNA by ORNi-PCR followed by real-time PCR

Next, we examined the utility of ORNi-PCR followed by ddPCR or real-time PCR for detecting *KRAS* mutations. Each dual-labeled probe designed to specifically detect *KRAS* mutations was first evaluated by ddPCR or real-time PCR. As shown in Supplementary Fig. S2, specific detection of *KRAS* G12D/V/C and G13D was achieved using a temperature of 60°C for the annealing plus elongation step by ddPCR. In the *KRAS* G12D detection, >100 false positive droplets (“rain”) were observed between the positions of positive (*KRAS* G12D) and off-target (*KRAS* WT) droplets (marked by an orange square in Supplementary Fig. S2) even at 60°C, indicating that additional modifications of the experimental conditions may be necessary. Next, we evaluated dual-labeled probes for detecting *KRAS* G12V/C and G13D by real-time PCR. These *KRAS* mutations were specifically detected at 64°C in the annealing plus elongation step (Supplementary Fig. S3). Because of the high frequency of the *KRAS* G12V mutation in PDAC and the detection specificity of the probes, detection of *KRAS* G12V DNA was used to examine the utility of ORNi-PCR followed by real-time PCR or ddPCR.

**Figure 4.**
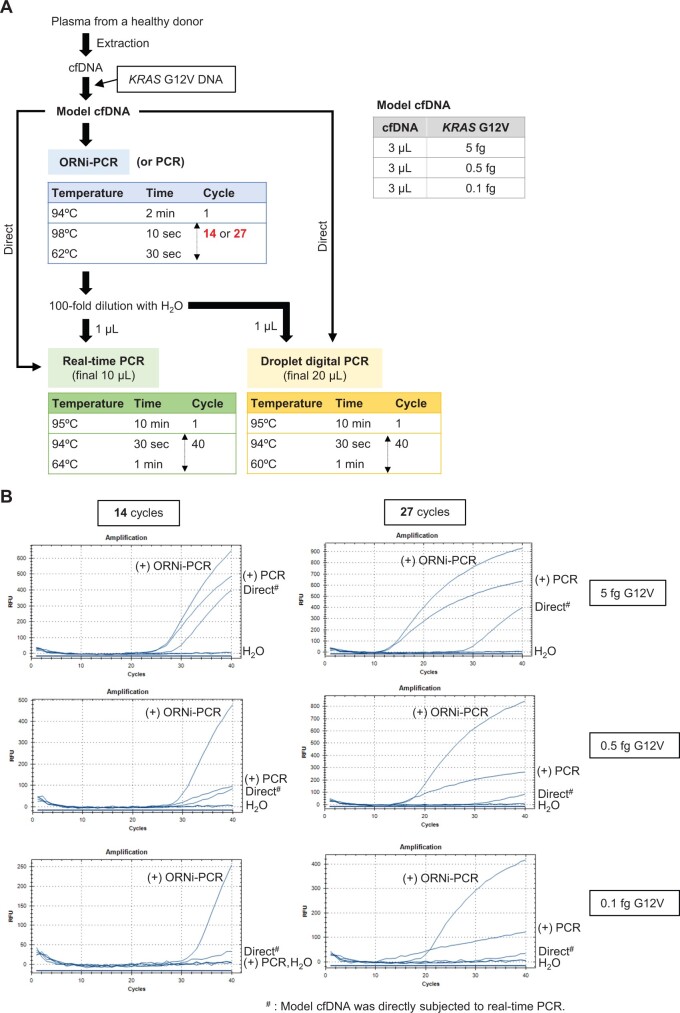
Detection of *KRAS* G12V DNA in model cell-free DNA (cfDNA). (**A**) Experimental strategy for the detection of G12V DNA mixed in cfDNA by ORNi-PCR followed by real-time PCR or ddPCR. Approximately 1 × 10^3^, 1 × 10^2^, and 2 × 10 copies of *KRAS* G12V DNA are contained in 5, 0.5, and 0.1 fg, respectively. (**B**) Results of ORNi-PCR followed by real-time PCR.

Given the potential application of the method to liquid biopsy, model cfDNA was used as the template for the ORNi-PCR-based methods. For this purpose, cfDNA was extracted from the plasma fraction of a healthy donor and mixed with *KRAS* G12V DNA to generate model cfDNA (Fig. 4A). This model cfDNA was first used for ORNi-PCR followed by real-time PCR to detect G12V DNA. Different numbers (14 and 27) of heating and annealing/elongation cycles of ORNi-PCR were tested (Fig. 4A). ORNi-PCR followed by real-time PCR detected 5–0.1 fg (ca. 1 × 10^3^–2 × 10 copies) of G12V DNA regardless of the number of cycles of ORNi-PCR (Fig. 4B). We also compared the efficacy of conventional PCR with that of ORNi-PCR (Fig. 4A). The use of 14 cycles did not clearly enhance detection of ≤0.5 fg of G12V DNA (Fig. 4B), whereas 27 cycles resulted in increased but weak fluorescent signals with ≤0.5 fg of G12V DNA (Fig. 4B). Amplification of endogenous *KRAS* WT DNA in cfDNA by conventional PCR may have prevented the amplification of the spiked-in G12V DNA, which could have caused the weak fluorescent signal detected by real-time PCR. These results support the higher accuracy of ORNi-PCR. Conventional real-time PCR using the cfDNA model (i.e. no ORNi-PCR/PCR prior to real-time PCR) failed to detect ≤0.1 fg of G12V DNA (“Direct” in Fig. 4B). These results suggest that ORNi-PCR followed by real-time PCR is more sensitive and accurate for the detection of *KRAS* mutant DNA in cfDNA.

### Detection of *KRAS* G12V DNA by ORNi-PCR followed by ddPCR

**Figure 5.**
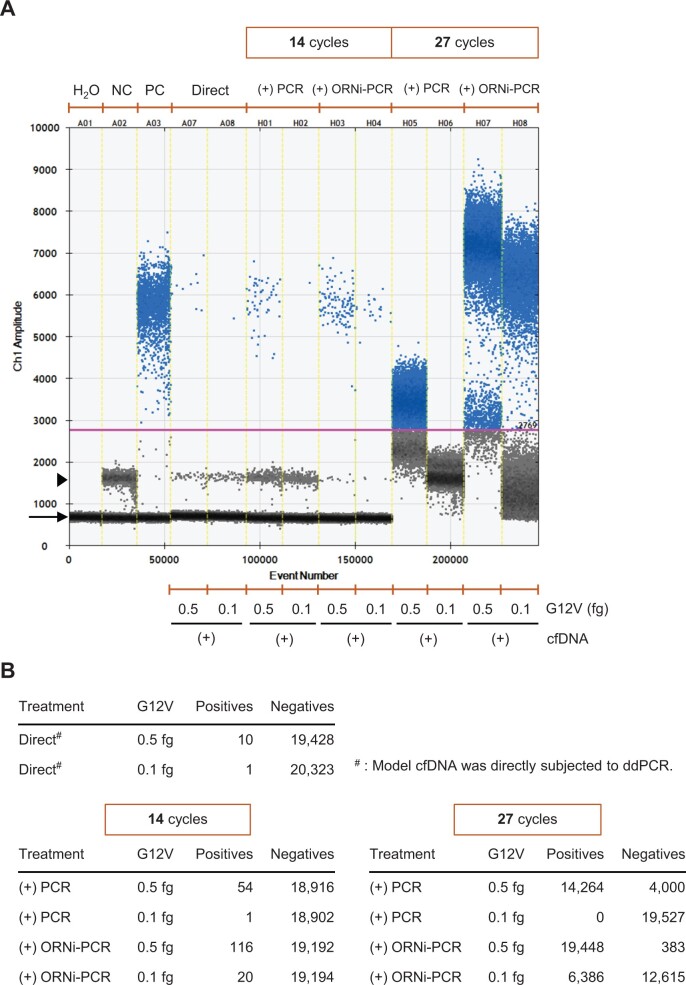
Detection of *KRAS* G12V DNA in model cfDNA by ORNi-PCR followed by ddPCR. (**A**) Results of ORNi-PCR followed by ddPCR. The positions of empty and potential off-target droplets including amplified *KRAS* WT DNA, primer dimers, and/or non-specific amplicons are shown by an arrow and arrowhead, respectively. NC: 50 fg of *KRAS* WT DNA; PC: 50 fg of *KRAS* G12V DNA. (**B**) Number of droplets counted. Droplets above and under the threshold line (purple) were counted as positive and negative droplets, respectively.

Next, we examined the efficacy of ORNi-PCR followed by ddPCR (Fig. 4A). We found that ddPCR of model cfDNA including 0.1 and 0.5 fg of G12V DNA detected 1 and 10 copies of positive droplets, respectively (“Direct” in Figs. 4A and 5). The number of positive droplets was 20–10-fold smaller than the expected copy number of spiked-in G12V DNA (2 × 10 and 1 × 10^2^ copies, respectively). This inconsistency may be caused by degradation or loss of DNA during experimental handling. Therefore, we considered the number of the detected positive droplets (1 and 10 droplet(s) with 0.1 and 0.5 fg, respectively) as the actual input copy number in this experiment. When 14 cycles of ORNi-PCR were applied to model cfDNA including 0.1 and 0.5 fg of *KRAS* G12V DNA before ddPCR, 20 and 116 positive droplets were detected by ddPCR, respectively (Fig. 5A and B). Because 1 and 10 copies of a target DNA are theoretically detected as 1.6 × 10 and 1.6 × 10^2^ positive droplets by ddPCR following 14 cycles of ORNi-PCR, respectively (Supplementary Fig. S4), the numbers (20 and 116) of detected positive droplets are reasonable. By contrast, when 14 cycles of conventional PCR were applied to cfDNA including 0.1 fg of *KRAS* G12V DNA before ddPCR, only one positive droplet was detected, which was comparable with the result of direct ddPCR (i.e. no ORNi-PCR/PCR prior to ddPCR) (Fig. 5A and B). When cfDNA including 0.5 fg of the *KRAS* G12V DNA was subjected to 14 cycles of conventional PCR before ddPCR, 54 positive droplets were detected (Fig. 5B), which was approximately one-half of the number of positive droplets of ddPCR detected following 14 cycles of ORNi-PCR.

When 27 cycles of ORNi-PCR were applied to cfDNA including 0.1 and 0.5 fg of G12V DNA before ddPCR, ˃1,000 positive droplets were detected (Fig. 5B). By contrast, ddPCR following 27 cycles of conventional PCR failed to detect 0.1 fg of the DNA (Fig. 5B). In addition, although ddPCR following 27 cycles of conventional PCR detected 0.5 fg of G12V DNA, the boundary between the positive and potential off-target droplets was ambiguous. These results indicate that a strategy to amplify a small amount of G12V DNA by conventional PCR prior to ddPCR is not feasible for amplification of the mutant signals by ddPCR.

Taken together, the results indicate that ORNi-PCR followed by ddPCR is a more sensitive and accurate method than direct ddPCR alone or ddPCR following conventional PCR for detecting small amounts of *KRAS* mutant DNA in cfDNA. In addition, the use of a larger number of cycles (e.g. 27 cycles) of ORNi-PCR is more effective for detecting the presence of *KRAS* mutant DNA unambiguously. One copy of target DNA is theoretically detected as 1.3 × 10^5^ positive droplets by ddPCR following 27 cycles of ORNi-PCR (Supplementary Fig. S4). When ddPCR was performed with 2.2 × 10^4^ droplets (Supplementary Table S4), all droplets were saturated with the amplified target DNA (5.9 copies/droplet) and detected as positive signals. However, ddPCR following 27 cycles of ORNi-PCR detected negative droplets, which accounted for two-thirds of total droplets. Thus, amplification of target DNA would be less effective than expected.

## Conclusions and limitations

In this study, we demonstrated the utility of ORNi-PCR followed by real-time PCR/ddPCR for detecting *KRAS* mutant DNA in cfDNA. The ORNi-PCR-based methods can detect low amounts of target mutant DNA with specificity and accuracy. ORNi-PCR followed by either real-time PCR or ddPCR detected 0.1 fg of G12V DNA (Figs. 4B and 5), indicating that the detection sensitivity and accuracy of the two methods are comparable. However, ORNi-PCR followed by real-time PCR is more cost- and time-effective. The ORNi-PCR-based methods are useful for detecting *KRAS* mutations in various types of cancer (e.g. colon cancer, lung cancer [11]), as well as for detecting other nucleotide mutations. Work from our group demonstrated that the ORNi-PCR-based methods can detect *anaplastic lymphoma kinase* gene mutations by liquid biopsy [12].

Future studies are necessary to assess the efficacy of ORNi-PCR-based methods for detecting *KRAS* mutations in PDAC patients by liquid biopsy. However, the present study had several limitations that need to be overcome. First, we only demonstrated detection of *KRAS* G12V DNA as a representative example in this study. Detection of other *KRAS* mutations using model cfDNA needs to be tested. Second, considering the average length of cfDNA (c.a., 0.17 kbp) [13], the amplicon size of ORNi-PCR (0.22 kbp, Supplementary Fig. S1) should be shorter. Therefore, the design of a primer set for amplifying a shorter amplicon would be beneficial. Pancreatic cancer has a poor prognosis because it is difficult to detect in the early stages and because of its high frequency of recurrence. In addition to their utility for detecting *de novo* pancreatic cancer, the ORNi-PCR-based methods could be of value for detecting pancreatic cancer recurrence through periodic monitoring by liquid biopsy after clinical treatment.

## Supplementary Material

bpae071_Supplementary_Data

## Data Availability

All data generated or analyzed during this study are included in this published article and the supplementary information.

## Supplementary data

*Conflict of interest statement.* T.F. and H.F. hold the following patents and patent applications for ORNi-PCR: (i) name: Method for suppressing amplification of specific nucleic acid sequences; patent numbers: Japan 6,928,323 and 6,964,900; (ii) name: Method for detecting differences in target nucleic acid region; patent publication number: WO 2019/203350; patent numbers: Japan 6,653,932, Canada 3,096,462, Korea 10-2,285,085, Israel 277,764, USA 11,155,873, and Singapore 11,202,009,878 V. Wisteriagen LLC owns the rights to the commercial use of ORNi-PCR. T.F. and H.F. are founders and directors of Wisteriagen, LLC.
