# Supplementary material for: Oligoribonucleotide interference-PCR-based methods for the sensitive and accurate detection of KRAS mutations
Source: Biol Methods Protoc. 2024 Oct 1;9(1):bpae071. doi: 10.1093/biomethods/bpae071 (PMC11522869; doi:10.1093/biomethods/bpae071)
Supplement: bpae071_Supplementary_Data [file bpae071_supplementary_data.pdf]

Oligoribonucleotide interference-PCR-based methods for the sensitive and accurate detection of KRAS mutations

Hiroaki Fujita[1], Toshitsugu Fujita[2], Keinosuke Ishido[1], Kenichi Hakamada[1], Hodaka Fujii[2]

1. Department of Gastroenterological Surgery, Hirosaki University Graduate School of Medicine, 5 Zaifu-cho, Hirosaki, 036-8562 Aomori, Japan
2. Department of Biochemistry and Genome Biology, Hirosaki University Graduate School of Medicine, 5 Zaifu-cho, Hirosaki, 036-8562 Aomori, Japan

Supplementary Table S1. Oligonucleotides used in this study

Supplementary Table S2. Components of a reaction mixture for ORNi-PCR

Supplementary Table S3. Components of a reaction mixture for real-time PCR

Supplementary Table S4. Components of a reaction mixture for ddPCR

Supplementary Figure S1. Template DNA including the human *KRAS* sequence. The plasmid pCR4_hKRAS_WT contains the *KRAS* WT sequence (WT DNA), whereas pCR4_hKRAS_G12D, pCR4_hKRAS_G12V, pCR4_hKRAS_G12C, and pCR4_hKRAS_G13D possess the sequences corresponding to G12D, G12V, G12C, and G13D, respectively. The plasmid pCR4_hKRAS_WT is shown as a representative plasmid. Primer positions are highlighted. The positions of G12 and G13 are indicated in blue and red bold font, respectively.

Supplementary Figure S2. Optimization of ddPCR for the specific detection of *KRAS* mutant DNA. (**A**) Experimental conditions for ddPCR with a dual-labeled probe. (**B**) Results of ddPCR for specifically amplifying *KRAS* mutant DNA. The “rain” of the *KRAS* probes at 60℃ is indicated in orange squares. The *KRAS* G12V/C probes show a wider amplitude between the positions of off-target (WT) and probe-specific droplets and a lower amount of “rain”.

Supplementary Figure S3. Optimization of real-time PCR for the specific detection of *KRAS* mutant DNA. (**A**) Experimental conditions for real-time PCR with a dual-labeled probe. (**B**) Results of real-time PCR for specifically amplifying *KRAS* mutant DNA.

Supplementary Figure S4. Expected number of positive droplets of *KRAS* G12V DNA by ddPCR following ORNi-PCR.

Supplementary Table S1. Oligonucleotides used in this study

Types	Numbers	Names	Sequences (5' to 3')
ORN	R72	ORN_KRAS_G12G13	ggagcugguggcguagg
Primers	28244<br>28245<br>28685<br>28686	hKRAS-F<br>hKRAS-R<br>hKRAS-G12G13-F<br>hKRAS-G12G13-R	GTGGGGGTCCACTAGGAAAACT<br>CCAATCAAAATGCACAGAGAGTG<br>GGTGAGTTTGTATTAAAAGGTACTGG<br>ATTGTTGGATCATATTCGTCCAC
Probes	28792<br>28881<br>28882<br>28883	Probe_KRAS_G12G13_sense2 (G12D)<br>Probe_KRAS_G12V_sense<br>Probe_KRAS_G12C_sense<br>Probe_KRAS_G13D_sense	FAM-TTGGAGCTGATGGCGTAGG-BHQ1<br>FAM-TTGGAGCTGTTGGCGTAGG-BHQ1<br>FAM-TTGGAGCTTGTGGCGTAGG-BHQ1<br>FAM-TTGGAGCTGGTGACGTAGG-BHQ1

Supplementary Table S2. Components of a reaction mixture for ORNi-PCR

	μL	Final concentration
H_2O	4.1–2.6	-
10×PCR Buffer (KOD -Plus- Ver.2)*	1.0	-
dNTPs (2 mM)*	1.0	0.2 mM
$MgSO_4$ (25 mM)*	0.6	1.5 mM
Primer F (10 μM)	0.3	300 nM
Primer R (10 μM)	0.3	300 nM
KOD -Plus- (1 U/μL)*	0.2	0.2 U
ORN (10 μM)	0.5–2.0	0.5–2.0 μM
DNA sample	2	-
Final Volume	10	

*Components of KOD -Plus- Ver.2 (KOD-211, Toyobo, Osaka, Japan)

Supplementary Table S3. Components of a reaction mixture for real-time PCR

	μL	Final concentration
H_2O	3.2	-
THUNDERBIRD Probe qPCR Mix (2x)#	5	1x
Primer F (10 μM)	0.3	300 nM
Primer R (10 μM)	0.3	300 nM
Probe (10 μM)	0.2	200 nM
DNA sample	1	-
Final Volume	10	

#QPS-101 (Toyobo)

Supplementary Table S4. Components of a reaction mixture for ddPCR

	μL	Final concentration
H_2O	4.9	-
ddPCR Super mix for Probes (2x)**	10	1x
Primer F (10 μM)	1.8	900 nM
Primer R (10 μM)	1.8	900 nM
Probe (10 μM)	0.5	250 nM
DNA sample	1	-
Final Volume	20	

**1863026 (Bio-Rad)

The reaction mixture was partitioned into ~ 22,000 droplets with 70 μL of Droplet Generation Oil (1863005, Bio-Rad) and then subjected to thermal cycling.

Supplementary Figure S1

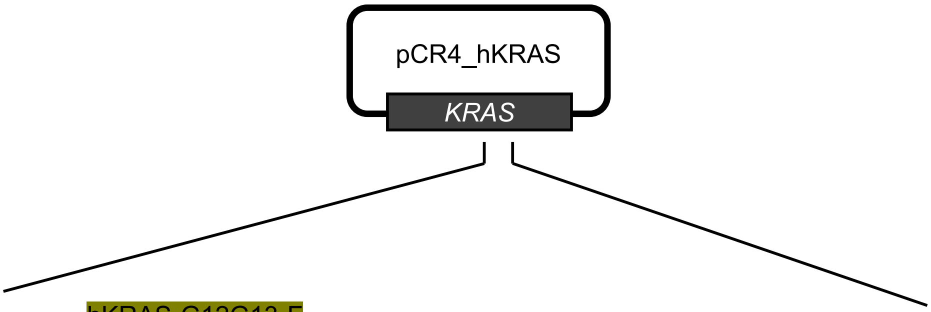

hKRAS-G12G13-F

5’…GGTGAGTTTGTATTAAAAGGTACTGGTGGAGTATTTGATAGTGTAT
TAACCTTATGTGTGACATGTTCTAATATAGTCACATTTTCATTATTTTTA
TTATAAGGCCTGCTGAAAATGACTGAATATAAACTTGTGGTAGTTGGA
GCT**GGTGGC**GTAGGCAAGAGTGCCTTGACGATACAGCTAATTCAGAA
TCATTTTGTGGACGAATATGATCCAACAAT…3’

hKRAS-G12G13-R

Supplementary Figure S2

A

Temperature	Time	Cycle
95ºC	10 min	1
94ºC	30 sec	40
54 - 60ºC	1 min	

Template DNA (50 fg each)

Plasmid DNA

- *KRAS* WT
- *KRAS* G12D
- *KRAS* G12V
- *KRAS* G12C
- *KRAS* G13D

B

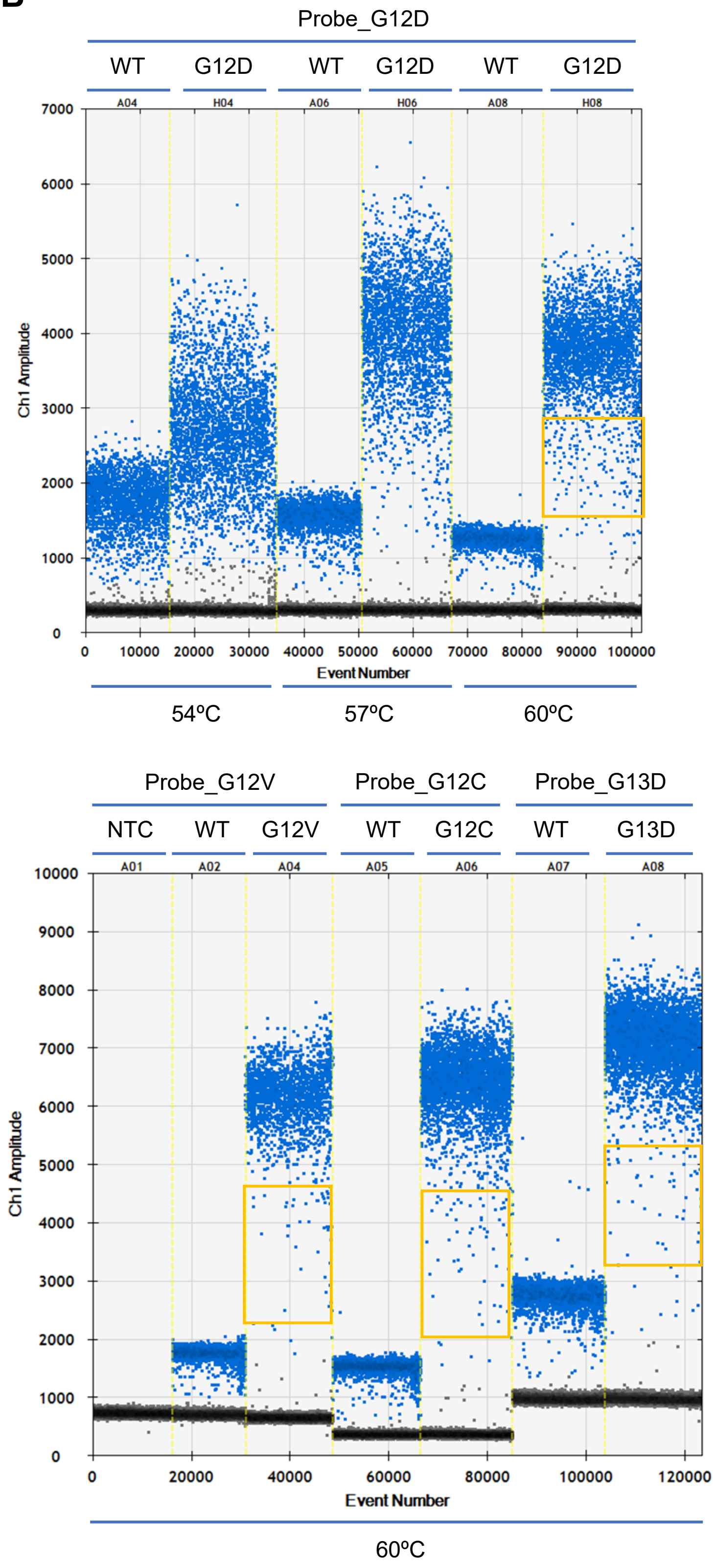

Supplementary Figure S3

A

Temperature	Time	Cycle
95ºC	10 min	1
94ºC	30 sec	40
64ºC	1 min	

Template DNA (50 or 0.5 fg)
Plasmid DNA
- *KRAS* WT
- *KRAS* G12V
- *KRAS* G12C
- *KRAS* G13D

B

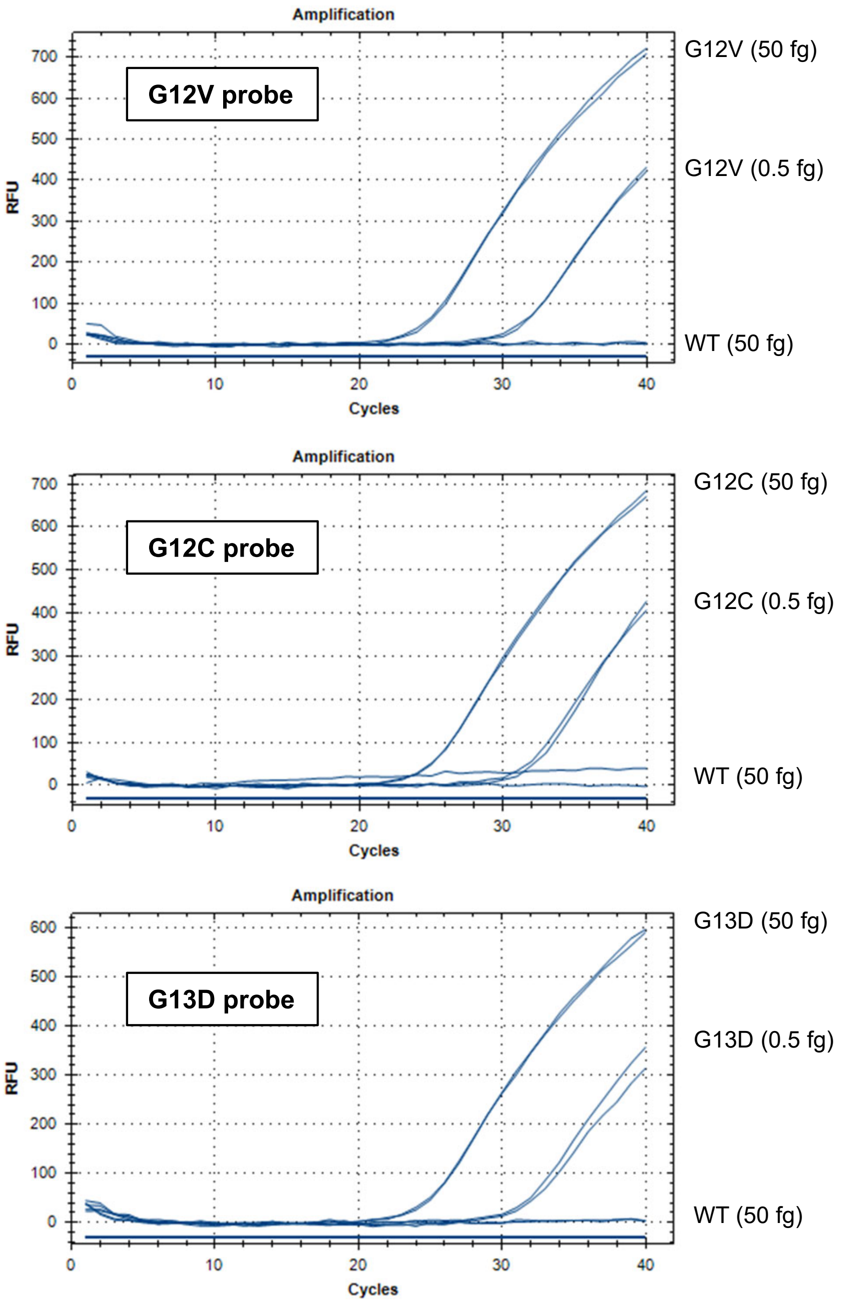

Supplementary Figure S4

Theoretical calculation

G12V	Input copy number		14 cycles of ORNi-PCR		100-fold dilution		1 μL subjected to ddPCR
0.5 fg	10 copies	→	10×2^{14} in 10 μL	→	10×2^{14} in 1,000 μL	→	$10 \times 2^{14}/1{,}000 = 1.6 \times 10^{2}$ copies
0.1 fg	1 copy	→	1×2^{14} in 10 μL	→	1×2^{14} in 1,000 μL	→	$1 \times 2^{14}/1{,}000 = 1.6 \times 10$ copies

G12V	Input copy number		27 cycles of ORNi-PCR		100-fold dilution		1 μL subjected to ddPCR
0.5 fg	10 copies	→	10×2^{27} in 10 μL	→	10×2^{27} in 1,000 μL	→	$10 \times 2^{27}/1{,}000 = 1.3 \times 10^{6}$ copies
0.1 fg	1 copy	→	1×2^{27} in 10 μL	→	1×2^{27} in 1,000 μL	→	$1 \times 2^{27}/1{,}000 = 1.3 \times 10^{5}$ copies

↑ Positive droplets detected in "Direct" in Figure 5

↑ Expected positive droplets
